# Crop Root Responses to Drought Stress: Molecular Mechanisms, Nutrient Regulations, and Interactions with Microorganisms in the Rhizosphere

**DOI:** 10.3390/ijms23169310

**Published:** 2022-08-18

**Authors:** Jian Kang, Yunfeng Peng, Weifeng Xu

**Affiliations:** 1 Division of Plant Science and Technology, University of Missouri, Columbia, MO 65211, USA; 2 Interdisciplinary Plant Group, University of Missouri, Columbia, MO 65211, USA; 3 State Key Laboratory of Vegetation and Environmental Change, Institute of Botany, Chinese Academy of Sciences, Beijing 100093, China; 4 College of Resources and Environment, Fujian Agriculture and Forestry University, Fuzhou 350002, China

**Keywords:** crops, drought, growth, nutrients, rhizosphere microorganisms, roots

## Abstract

Roots play important roles in determining crop development under drought. Under such conditions, the molecular mechanisms underlying key responses and interactions with the rhizosphere in crop roots remain limited compared with model species such as *Arabidopsis*. This article reviews the molecular mechanisms of the morphological, physiological, and metabolic responses to drought stress in typical crop roots, along with the regulation of soil nutrients and microorganisms to these responses. Firstly, we summarize how root growth and architecture are regulated by essential genes and metabolic processes under water-deficit conditions. Secondly, the functions of the fundamental plant hormone, abscisic acid, on regulating crop root growth under drought are highlighted. Moreover, we discuss how the responses of crop roots to altered water status are impacted by nutrients, and vice versa. Finally, this article explores current knowledge of the feedback between plant and soil microbial responses to drought and the manipulation of rhizosphere microbes for improving the resilience of crop production to water stress. Through these insights, we conclude that to gain a more comprehensive understanding of drought adaption mechanisms in crop roots, future studies should have a network view, linking key responses of roots with environmental factors.

## 1. Introduction

Drought stress is one of the most serious abiotic stresses affecting plant growth and crop productivity [1,2]. In recent years, crop production has become water-limited and will be more affected in the future due to climate change worldwide [3,4]. Under such circumstances, the ability to survive under drought stress is critical for the growth and survival of crop species [5,6]. Therefore, research into how crops respond to exposure to soil water deficit is essential for producing high-yield and drought-tolerant crops.

Among the plant organs that are affected by water-deficit stress, the root is the first organ that emerges from the seed and contacts the rhizosphere [7,8,9]. Water and nutrients are absorbed by roots and then transported to the rest of the plant [8,10]. Shortage of soil water content leads to water deficiency in the plant rhizosphere. When sensing water deficits, crop roots transmit stress signals to the rest of the plant and cause developmental, physiological, and metabolic changes in adaptation to the drought stress. The study of crop root responses to drought stress is important for clarifying how crops can maintain growth under water-deficit conditions and provides candidate genes for genetic manipulation to improve crop traits, drought tolerance, and ultimately, grain yield. Therefore, the establishment of the root system is essential for the development and survival of crops, especially when the seedling is exposed to drought [9,11], which can be critical for productivity and grain yield in an agricultural sense [12,13].

**Figure 1:**
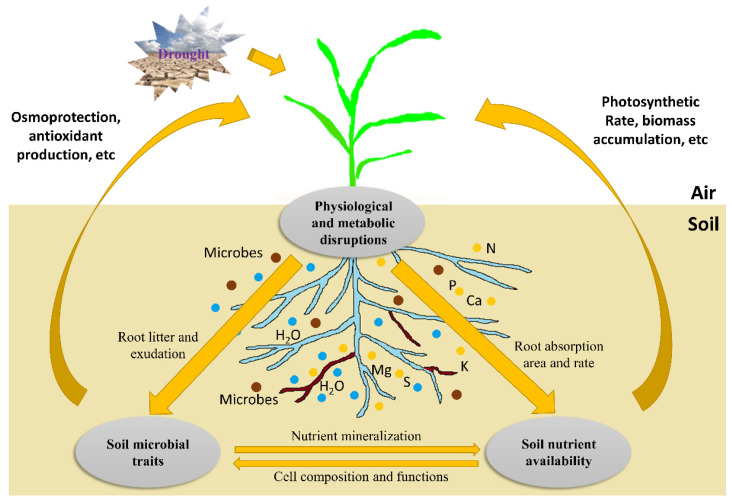
A framework showing drought effects on root physiological and metabolic processes and their interactions with nutrients and microbiomes in soil. Severe drought causes physiological and metabolic disruptions (see below for details). On the one hand, these disruptions influence nutrient uptake and metabolisms in plants, and vice versa. This feedback, in turn, affects biomass accumulation and grain yield. On the other hand, the inhibition of root litter and exudation production alters soil microbial traits (e.g., abundance, composition, and activity). These soil microorganisms may interact with roots in the rhizosphere, promoting plant growth via mycorrhizae colonization, osmoprotection, antioxidant production, etc. Under drought stress, soil microbes also affect nutrient availability through mineralization, and nutrients influence microbial growth via participation in their cell composition and functioning. Blue circles represent water resources in soil; yellow circles represent nutrient resources in soil; brown circles represent microorganisms in soil.

The responses of plant roots to water-deficit stress have been the focus of research in recent decades [6,11,14,15]. When exposed to drought, the metabolism and physiological processes in plants are disturbed [6,16]. As the organ first sensing water deficiency, roots sense the stress soon after the exposure and generate specific responses to the stress. Physiologically, the plant–water relations are changed, leading to a shift in some metabolic pathways. The allocation of photosynthate in roots and the rhizosphere is inhibited under severe drought conditions. Thus, the inhibition of root growth will in turn reduce water and nutrient uptake, ultimately influencing both biomass accumulation and grain yield. Under water-deficit conditions, a well-developed root system architecture (RSA) can enhance the drought tolerance of crops and improve the utilization of resources, thereby increasing the yield and quality of crops. With optimized RSA, crops can strengthen their traits, including depth and root number, to better absorb deeper soil water resources to improve their drought tolerance [17,18,19,20]. Therefore, RSA characteristics have become important considerations for crop breeders [21,22]. Plant hormones play important roles in roots through interactions between hormones and with metabolites. Abscisic acid (ABA) serves as a root-generated signal to regulate the opening of stomata and wilting of leaves in the upper parts of crops to resist water-deficit stress, providing a theoretical basis for improving water-use efficiency (WUE) by regulating water deficiency status in the rhizosphere [23,24,25]. It is one of the most studied plant hormones in recent years, especially for its regulatory roles in roots [26,27] and interactions with other hormones [28,29,30]. Meanwhile, studies have shown that crop root response to drought is regulated by nutrients. For example, the application of nutrients such as nitrogen under water-deficit conditions can improve the responses of roots to drought and change RSA for increased drought tolerance [31]. Crop responses to water-deficit stress are also affected by rhizosphere microorganisms. Changes in root exudation will alter the abundance, composition, and activities of rhizosphere microorganisms, as well as the feedback between crops and soil microbes. Root exudation may also enhance the activities of nutrients and water uptake from roots (Figure 1).

A large number of studies have been conducted to investigate the mechanisms that underlie the morphological, physiological, and metabolic responses to drought stress in roots [32,33,34,35,36,37]. Importantly, several systematic reviews have also been published to document how plant roots respond to drought [9,15,38,39]. To date, however, a comprehensive understanding of the mechanisms underlying root responses to drought and the interactions between roots and the surrounding environment (particularly interactions with nutrients and soil microbiomes and how these interactions regulate the root responses to water-deficit stress) in major crops has been mainly focused on one or two model crops, and the number of reviews targeting crops with respect to this topic remains limited compared with the number of reviews on *Arabidopsis*. In this review, we highlight recent advances that address the following aspects: (1) morphological changes in the growth and architecture of crop roots in response to drought stress; (2) metabolic mechanisms regulating the adaption to drought stress in crop root cells; (3) regulatory roles of ABA and interactions between ABA and other plant hormones in crop roots; (4) how roots sense and respond to drought stress, as regulated by the availability of nutrients; and (5) drought-induced regulation of rhizosphere microbes with crop roots. With the background of climate change and water resource limitations across the world’s agricultural areas, a better understanding of these mechanisms and processes could contribute to the establishment of ideal RSA adaptation to drought by exploring key genes for the drought tolerance of roots. At the same time, through the regulation of water and nutrient resources as well as rhizosphere microbes in the root area of the field, it is possible to reduce fertilization and irrigation and thus improve the efficiency of resource utilization in order to achieve the goal of sustainable crop production by producing water-saving, drought-tolerant, and high-yield crops under drought conditions.

## 2. Crop Root Growth and Architecture in Response to Drought Stress

### 2.1. Root Growth

Under drought conditions, root length, diameter, surface area, tissue density, biomass, and number of branches are affected [40,41,42]. However, roots have strategies to maintain functions and grow toward a more mesic environment to maintain water uptake under circumstances where soil water availability is limited and overall growth is inhibited [43,44,45,46]. Therefore, even though water-deficit stress negatively affects overall root traits in many circumstances (i.e., root biomass, root length, root number, etc.), certain parts of the root system can maintain growth to form a relatively well-developed root system under drought conditions for acquiring potential water resources from a deeper soil layer to ensure water supply and improve the adaptation of crops to drought stress. To achieve such an ideal root system, the primary root, the very first root tissue that emerges from the seed and makes contact with the environment, can maintain growth, relative to shoot growth, by maintaining cell elongation in the apical regions of several crops, including maize, soybean, and cotton, under relatively low water content conditions (−1.0~−1.6 MPa media water potentials) [37,47,48,49]. The underlying mechanisms of primary root maintenance include accumulation of osmolytes, hormone regulations, cell-wall modification, and specialized regulation of antioxidative systems [35,37,49,50,51,52,53,54,55,56,57]. However, specific molecular regulations involved in this process in crops require further investigation. As the other part of root systems, lateral roots constitute the major portion of the root system [58]. Because of the contact of numerous branched lateral roots with the soil, lateral roots are essential for water acquisition [59,60]. In many cases, lateral root growth is limited under water-deficit conditions [41,61,62]. However, the formation of lateral roots can be promoted under water-deficit conditions by stimulating the growth of a certain composition of the root system [63,64,65].

**Figure 2:**
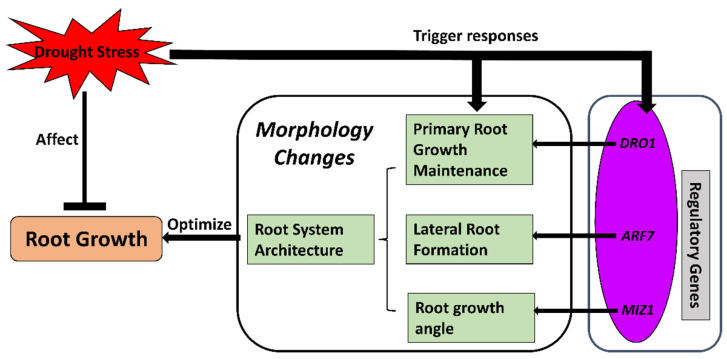
Morphological changes occurring in crop roots under drought stress. Drought stress prevents root growth while triggering regulatory mechanisms to change root morphology. Regulatory genes are activated to modify the root traits for better root structure architecture (RSA) under drought stress. Optimized RSA could maintain or promote root growth under such conditions. *ARF7*, AUXIN RESPONSE FACTOR 7 gene; *DRO1*, DEEPER ROOTING 1 gene; *MIZ1*, MIZU KUSSEI 1 gene.

With the morphological findings of adjusted root growth characteristics under drought, more-recent studies on crop roots under water-deficit conditions have mainly focused on how the transitions of whole root system characteristics are regulated by certain genes or molecular mechanisms. Among these mechanisms, root hydrotropism, lateral root growth, and deeper rooting are three essential aspects of root growth and root system formation that have been focused on with molecular approaches in recent years (Figure 2).

As a gene that is essential for hydrotropism to regulate the angle of roots [66,67], MIZU KUSSEI1 (*MIZ1*) has been studied in *Arabidopsis* and found to regulate root hydrotropism by encoding a protein associated with the cytoplasmic side of the endoplasmic reticulum membrane. It interacts with the endoplasmic reticulum Ca^2+^–ATPase (*AtECA1*) and modulates hydrotropism-related Ca^2+^ transport [66,68,69,70]. *MIZ1* has also been found to interact with ABA signaling [71,72] and relate to root gravitropism [73]. In crops, the MIZ1 gene *OsMIZ1* has been cloned and characterized in rice roots. *OsMIZ1* expression levels were upregulated significantly in the roots of drought-tolerant rice lines but decreased in the roots of susceptible genotypes [74]. However, it has not been investigated in other major crops apart from a brief mention in a preliminary maize study [75].

Another key gene for root formation regulation is AUXIN RESPONSE FACTOR 7 (*ARF7*), which mainly regulates the initiation of lateral roots through hydropatterning, the process of root branching into areas with higher water content [76,77]. *ARF7* is SUMOylated (modified by small ubiquitin-like modifier (SUMO)) in the area phase of the root and repressed by IAA3 (indole-3-acetic acid) [77,78]. Repressed *ARF7* prevents the expression of lateral root initiation genes, and therefore, lateral roots are formatted in the moisture phase that triggers hydropatterning [77]. In crops, *ARF7* has been identified in *Sorghum* (*SbARF7*) with high expression levels in roots in contrast with leaves under drought conditions [79]. However, the roles of *ARF7* in regulating lateral root growth in other crops under such conditions have not been completely uncovered.

As for genes regulating the maintenance of root growth, DEEPER ROOTING 1 (*DRO1*), a quantitative trait locus controlling root growth angle, has been identified and mainly studied in rice for its role in forming deeper roots under drought conditions [80,81]. The alleles of *DRO1* and its homologs can increase the gravitropism of rice roots, which contributes to drought stress avoidance [82,83]. Even though the whole picture of *DRO1* regulation in root formation is not clear at present, it possibly contributes to the establishment of an auxin gradient in the root tips since it is negatively regulated by auxin [84,85]. In genotypic variety studies, the *DRO1* gene was found to be significantly correlated with the drought tolerance of rice genotypes as well as the activities of antioxidative enzymes [86]. An allele mining study revealed that *DRO1* expression was upregulated under drought conditions in various rice genotypes [87]. These findings could be useful for breeding programs identifying drought-tolerant rice lines. Other than rice, *DRO1* homologs have been identified and studied in several plant families recently, including *Arabidopsis* and *Prunus*, with evidence of promoting deeper rooting and lateral root angle [88]. However, even though orthologs of *DRO1* have been found in a variety of crops, including maize and tomato [88], it has not been well-studied in other crop roots for potential comparison with current findings in rice for possible general mechanisms of *DRO1* regulation in crop roots.

Additionally, several other genes and proteins have been shown to influence root growth or formation under drought conditions, but with less knowledge about their molecular regulatory mechanisms. The overexpression of plant-specific transcription factors NAM, ATAF1/2, and CUC2 (*NACs*) was found to regulate root growth by increasing lateral root length and number in transgenic *Arabidopsis* lines [89] and some of the *NAC* family transcription factors specifically expressed in soybean roots under water-deficit conditions [90,91]. *NACs* also participate in the modification of root architecture by changing root diameter and root numbers under drought stress in rice [32,42]. In rice, REPETITIVE PRO-RICH PROTEIN (RePRP), an intrinsically disordered protein functioning as a flexible stress modulator, can interact with the cytoskeleton to regulate root formation in rice in adaptation to water-deficit stress [92]. In maize, overexpression of *ZmVPP1*, a gene encoding vacuolar-type H^+^ pyrophosphatase, a unique electrogenic proton pump in plants, conferring drought-inducible expression in drought-tolerant maize genotypes, exhibits improved tolerance in a transgenic line, which is most likely due to enhanced root development. The transfer of *ZmVPP1* into drought-sensitive lines can effectively promote their drought tolerance in the seedling stage [93].

As summarized above, the ability to maintain root growth as soil water content declines is reported in various types of crop roots. This is an important characteristic of plant roots when resources are insufficient under drought conditions. From the studies mentioned above, it is notable that among these key genes regulating root growth and tropisms under water-deficit conditions with a clear mechanistic explanation, very few studies have been fully conducted in crops compared with the more completed studies on the model species, *Arabidopsis*, or in a specific crop species (e.g., *DRO1* in rice), even though the genes have been identified in other crops. This is a key perspective that should garner more attention in crop research for uncovering the molecular regulatory mechanisms of how crop roots respond to drought stress. In addition, the roles of these genes in different root types also need further clarification since the responses of different root types (e.g., primary root or lateral root) vary substantially under drought conditions.

### 2.2. Root System Architecture

For crops, the eventual purpose of regulating root growth characteristics is to optimize the RSA, which is a more complex strategy in response to drought stress (Figure 2). RSA consists of the architecture of primary and lateral roots, where lateral root architecture is a key factor of water and nutrient uptake from soil. Lateral root growth and branching are determined by the availability of water around the perimeter axis of the root [76,94] and are regulated by various genes, as summarized above. Root tropisms, including hydrotropism and xerotropism, are also key aspects of RSA by orientating root growth (mainly in lateral roots) under water-deficit conditions. Hydrotropism, regulated by *ARF7* or *MIZ1* as summarized above, may sense and orientate root growth towards soil water resources in any direction [95]. Xerotropism could enhance the ability of roots to grow downwards to avoid dry soil layers, orientated by gravitropism under water-deficit conditions [38], which can be regulated by ABA-induced auxin reduction to inhibit lateral root formation [94] on the aerial gaps in the soil. The plant modifies the RSA actively in response to different environmental stimuli [96,97]. Under such environmental conditions, including drought, better RSA can improve the utilization of limited resources and thus improve the yield and quality of crops. Therefore, recent studies have started to investigate the regulation of RSA under drought conditions. Typically, RSA is determined by root density, root distribution, and the anatomical characteristics of root structure. In crops, it has been reported that soybean genotypes with deeper, longer, denser, and narrower roots have better adaptation to drought [20]. Venuprasad et al. [98] reported that by analyzing the correlation of rice yield and the genotypic and phenotypic parameters of roots, it was found that genotypes with thicker and deeper roots produced more biomass and grain yield, which improved the production of rice under drought. When studying the adaptation of rice root morphological traits in the drought environment, Henry et al. [99] found that rice lines that have deeper and denser roots in 30–45 cm soil layers presented better drought tolerance. Therefore, deeper rooting improves the drought tolerance of rice, which can contribute to better crop productivity. Lopes and Reynolds [17] also found a positive correlation between the depth of wheat roots and yields under water-deficit conditions. By utilizing the relationship between root depth and crop drought tolerance, breeding projects in India and Australia chose deep-rooting wheat lines in order to maximize the uptake of summer rainwater stored deep in the soil. The RSA characteristics of drought-tolerant maize varieties having greater root lengths and lateral root numbers resulted in the production of more seeds, which significantly differed from drought-sensitive lines [19]. However, Zhan et al. [63] studied the relationship between maize root branches and drought tolerance and found that fewer lateral root branches are beneficial to improve the drought tolerance of maize. By reducing root density, maize roots can reduce the consumption of limited resources in total metabolism and provide more energy for the elongation of roots toward deeper soil layers, thus increasing the uptake and utilization of deep soil water resources via the root system. More interestingly, breeding selection found some wheat varieties used hybrid strategies to deal with root architecture. The overall trend of these root systems is to go deeper and have denser lateral roots in the deeper layers, while in the top layers, root density is low. This ideal RSA could both absorb water resources from deep soil layers and reduce the redundant consumption of limited resources under water-deficit conditions [18]. From the perspective of molecular breeding, obtaining drought-tolerant phenotype traits of crop roots is the foundation of identifying the key regulatory genes for optimized RSA and controlling the root architecture and functional characteristics of crops by allowing manipulation of key genes for breeding drought-tolerant and water-saving crop lines.

To easily obtain these phenotypes, advanced image technologies improve the accessibility of phenotyping the growth and distribution of root systems with more accurate 3D structures and spatial connection relationships of the crop root system in the rhizosphere. These technologies include X-ray computed tomography (XCT) [100], magnetic resonance imaging (MRI) [101], and laser ablation tomography (LAT) [102]. However, there are still many challenges on how to establish an ideal root system configuration through the study of root structure response to drought due to the complexity of the environment.

## 3. Regulation of Cellular Metabolism in Response to Drought Stress in Crop Roots

Based on the findings on root growth and architecture responses to drought stress, different aspects of cellular metabolic mechanisms involved in such responses have been the focus of various research. This section mainly focuses on three primary metabolism areas—osmotic regulation, cell-wall modification, and antioxidative mechanisms—based on their functions in regulating crop roots in response to drought stress, for which many studies have been conducted.

### 3.1. Osmotic Adjustment

**Figure 3:**
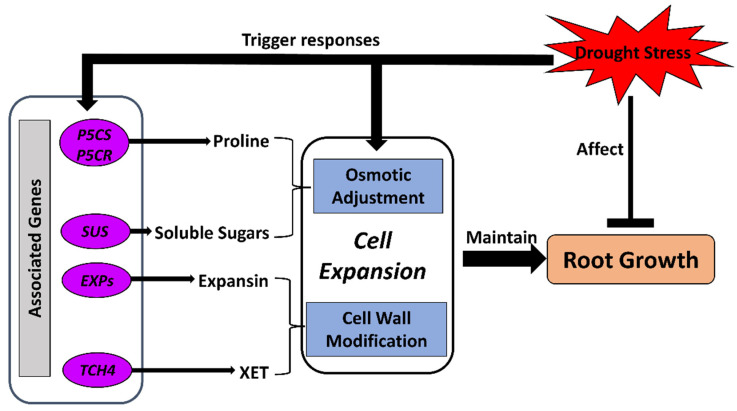
Osmotic adjustment and cell-wall modification processes for cell expansion in response to drought stress in crop roots. Drought stress limits root growth while triggering the regulatory mechanisms of an osmoprotectant. Regulatory genes are activated to accumulate osmolytes and modify cell-wall components for extensibility. The regulated cellular osmotic status and cell walls assist the expansion of root cells and, therefore, maintain growth of crop roots under drought stress. *EXPs*, EXPANSIN genes; *P5CR*, pyrroline-5-carboxylate reductase gene; *P5CS*, pyrroline-5-carboxylate synthetase gene; *SUS*, sucrose synthase gene; *TCH4*, xyloglucan:xyloglucosyl transferase gene.

Osmotic adjustment is one of the major mechanisms in response to drought stress regulating plant–water relations and maintaining root growth by regulating root cell expansion [50,51,103,104]. Cell expansion in roots is highly dependent on water availability and osmotic adjustment, which contribute to the maintenance of turgor in cells. When the water potential of the environment reduces, osmotic adjustment starts to regulate osmotic water potential in certain parts of the root system to maintain turgor for growth when shoot and leaf growth are repressed. The regulation of osmotic adjustment could affect the growth of different plant tissues, thus ensuring contact of the root system with possible water resources [48,50,64] (Figure 3).

To regulate osmotic adjustment, osmolyte accumulation has been recorded in crop roots under water-deficit conditions. Typically increased osmolytes include carbohydrates, normally soluble sugars [37,50,105,106], and the amino acid proline [34,37,51,107]. Additionally, studies have shown that transcripts of several key genes involved in sucrose metabolism (sucrose synthase gene (*SUS*)) and proline metabolism (pyrroline-5-carboxylate synthase gene (*P5CS*); pyrroline-5-carboxylate reductase gene, (*P5CR*)) were increased in abundance in water-stressed roots of crops, including soybean, maize, and wheat [92,108,109,110]. The accumulation of compatible solutes, including proline, participates in protecting the plants from the detrimental effects of drought. This is achieved not only by osmotic adjustment, but also by the detoxification of reactive oxygen species (ROS), protection of membrane integrity, stabilization of enzymes and/or proteins, and interactions with plant hormones [107,111,112]. Accumulation of proline specifically in cotton roots has been identified under water-deficit conditions [113]. Transgenic studies have shown that overexpression of the *Arabidopsis EDT1/HDG11* gene, a transcription factor that improves drought tolerance in *Arabidopsis*, tobacco, tall fescue, and rice, can enhance tolerance to drought stress in transgenic cotton with well-developed root systems through increased accumulation of solutes such as proline and soluble sugars, and thus increased the yield of cotton in the field [33]. Overexpression of Rice NAC Gene (*SNAC1*) in a cotton transgenic line can also increase the proline content, resulting in enhanced development of roots [114].

### 3.2. Cell-Wall Modification

As an important process of root growth and development, the regulation of cell-wall components and expansion are essential for adaptation to drought stress in crop roots. The expansion of plant cells is controlled by a combination of turgor pressure and the mechanical properties of the cell wall. As the roots grow, the development of the cell wall is modified by the availability of soil water to ensure the extensibility for further expansion. The root can continue to grow under water-deficit conditions with loosened cell walls and maintained turgor pressure in cells by regulators such as expansins (Figure 3). During the continuous expansion of plant roots under water-deficit conditions, expansins are considered key regulators of cell-wall extension during root growth, especially for growth maintenance [53,115]. As a classic group of cell-wall-remodeling agents, expansins are known to change the mechanical properties of the cell wall by disrupting noncovalent bonds in cell-wall polymers and creating local mechanical alterations in the cell wall [116]. Specifically, EXPANSIN A1 has been identified as a cell-wall modifier during lateral root initiation in *Arabidopsis* [117]. Increased abundance of expansin genes has been identified in maize primary roots [54] and rice lateral roots [118] under drought conditions.

Xyloglucan:xyloglucosyl transferase (XET) is a key enzyme in cell-wall modification that cleaves the (1,4)-β-d-glucan backbone of xyloglucans and transfers the bound portion of the initial substrate xyloglucan onto the end of another polysaccharide, which can be xyloglucan, xyloglucan-derived, or alternatively, xyloglucan-non-derived [119,120]. It contributes to cell-wall loosening and thus is utilized in restructuring and remodeling plant cell walls. In maize primary roots, XETs have been found to be increased in the apical root tip (0–5 mm) and were associated with ABA under water-deficit conditions [52]. Expression of the XET gene in cotton, *TCH4*, was also found to be highly increased in root tissues of drought-tolerant lines compared with sensitive lines under drought conditions [121].

Regarding the modification of cell-wall thickness, Henry et al. [122] reported that hydration thickening of cell walls in endodermis cells can increase to different extents among rice genotypes. Drought-resistant lines can store more water in their endodermis cells in response to water deficits. Rincon et al. [123] found that the endodermis appears to be a limiting barrier to water conductance, with dimensions relative to the exodermis varying among soybean genotypes. This indicates that the hydration thickening of cell walls can potentially affect root hydraulic conductivity. However, such relations were not found in *Arabidopsis* natural mutants [124]. Therefore, the relationship between hydration thickening of cell walls and root hydraulic conductivity may be unique to legume roots and requires further experiments.

Additionally, transcriptomic and proteomic studies display increased abundance of transcripts and enzymes related to cell-wall modification and cell-wall extension in roots of wheat [125] and cotton [126,127] genotypes that have better adaption to drought.

### 3.3. Antioxidants

**Figure 4:**
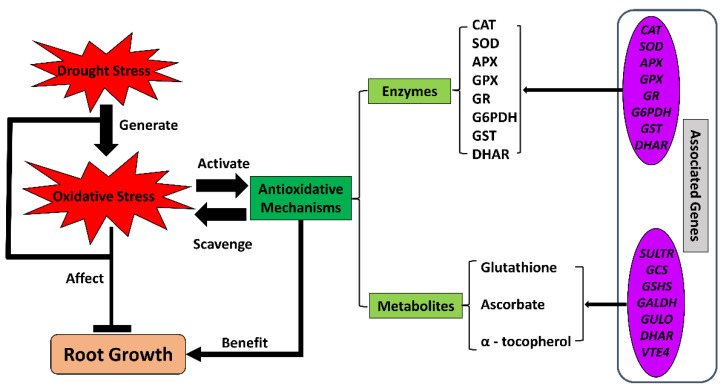
The antioxidative system under drought conditions in crop roots. Drought stress limits root growth while generating oxidative stress. Antioxidative mechanisms, including enzymes and non-enzymatic metabolites, respond to the oxidative stress by regulating the expression of a wide range of related genes. The activated antioxidative system scavenges the oxidative radicals and restores cellular homeostasis. APX, ascorbate peroxidase; CAT, catalase; DHAR, dehydroascorbate reductase; SOD, superoxide dismutase; G6PDH, glucose-6-phosphate dehydrogenase; *GALDH*, L-galactose dehydrogenase gene; *GCS*, gamma-glutamylcysteine synthetase gene; GPX, glutathione peroxidase; GR, glutathione reductase; *GSHS*, glutathione synthase gene; GST, glutathione S-transferase; *GULO*, L-gulonolactone oxidase gene; *SULTR*, sulfate transporter; *VTE4*, gamma-tocopherol methyltransferase gene.

Oxidative stress has a major impact on plant cells under drought conditions, as has been reported in multiple studies on crops. At the cellular level, ROS accumulate upon exposure to water-deficit stress and damage cellular homeostasis, causing protein denaturation, lipid peroxidation in cellular membranes, DNA or RNA damage, negative effects on antioxidative enzyme activities, and ultimately cell death [128,129]. Thus, the plant uses multiple antioxidants, including enzymes [130,131,132] and non-enzymatic metabolites [133,134,135] to resist oxidative damage (Figure 4).

The antioxidative functions of these enzymes and metabolites have been demonstrated widely in crop roots under drought conditions. In maize primary roots under severe water-deficit stress, transcriptomics and proteomics studies have identified increases in the abundance of several antioxidative enzymes and their transcripts in the growth zone, including catalase (CAT) and superoxide dismutase (SOD) [108,136]. Ascorbate and glutathione contents also increase in maize roots under water-deficit conditions [34,137]. In a comparative study of the antioxidative system in maize and wheat roots under drought, the antioxidants displayed differential accumulations in the two species. Antioxidative enzymes, including ascorbate peroxidase (APX), dehydroascorbate reductase (DHAR), and glutathione reductase (GR), as well as ascorbate and glutathione compounds, accumulated in maize roots under drought. In contrast, wheat root specifically possessed more activity of CAT compared with maize [138]. Another comparative study indicated that alternative antioxidative strategies underlie the responses of primary root growth to water stress between cotton and maize and that cotton employs a more enzyme-dependent and less glutathione-involved strategy in the elongation zone of primary roots [37]. A cytosolicglyceraldehyde-3-phosphate dehydrogenase, TaGAPC2, critical for glycolytic and gluconeogenesis metabolism, positively responded to water-deficit stress via ROS scavenging, and the encoding gene *TaGAPC2* was highly expressed in the root of wheat under water-deficit conditions [139]. In rice, the ERF (Ethylene Responsive Factor)-family transcription factor *OsLG3* was found to be expressed at higher levels in roots compared with other tissues. This transcription factor positively regulated drought tolerance by inducing the antioxidative system via upregulating the expressions of antioxidative enzyme genes under drought [36]. Several other studies regarding genotypic variations in root responses under water-deficit conditions identified the enhancement of antioxidative enzymes in drought-tolerant lines of multiple crops, including wheat [110,140,141], cotton [113,142], and barley [143].

**Figure 5:**
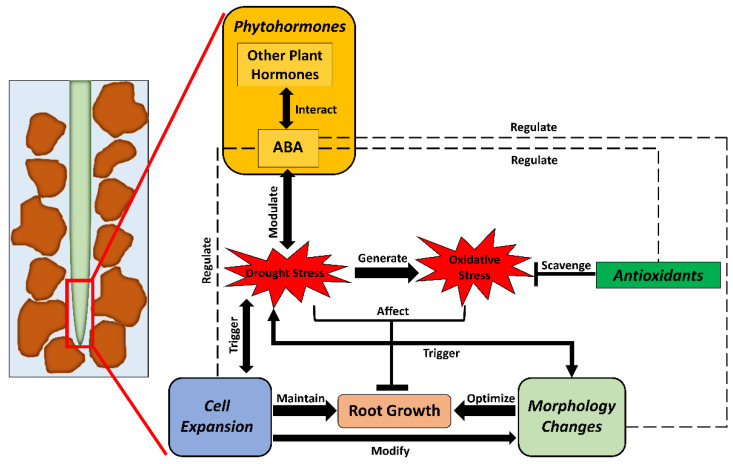
Overview of physiological and metabolic processes for growth maintenance of crop roots under drought stress. When exposed to drought, plant roots start to modify osmotic regulation to maintain turgor for continued root growth. Simultaneously, root morphological traits are changed to adapt to the lowering water status. Meanwhile, oxidative stress is generated in root tissue when cellular homeostasis becomes unbalanced. Under this circumstance, crop roots activate the antioxidative system rebalance cellular homeostasis. Plant hormones also modulate osmotic regulation and antioxidative mechanisms under drought conditions.

Overall, osmotic adjustment maintains cellular water balance and homeostasis in crop root tissues, which reduces the damage caused by water losses and maintains the turgor of root cells. It is a prerequisite for the loosened cell walls to expand with modifications from associated proteins. Simultaneously, antioxidants scavenge ROS radicals, which are generated with unbalanced cellular homeostasis. Together, these mechanisms increased the accessibility of crop roots to water resources with maintained or enhanced root growth (Figure 5). However, some studies have indicated that the changes in these mechanisms may vary among different crops (e.g., antioxidative mechanism in maize, wheat, and cotton). Future research should focus more on the uniqueness of these mechanisms as well as explore the specialized interaction network of these mechanisms in the roots of certain crop species under drought conditions.

## 4. Roles of ABA in Crop Roots under Drought

Plant hormone regulation is another important and complicated mechanism modulating plant growth under drought conditions. This article mainly summarizes the regulation of ABA, one of the key plant hormones with more-developed studies in regulating root growth in response to water-deficit stress and its interactions with several other plant hormones in crops.

### 4.1. Root-to-Shoot Communication

ABA is a well-known plant hormone that accumulates in water-stressed plant tissues, including roots under water-deficit conditions [144,145]. ABA can be produced in roots surrounded by dry soil and transported to the upper parts by transpiration. ABA is an important signaling molecule for root–shoot communication and regulation of stomatal closure [146], as well as root formation regulation. Since ABA signaling transduction is a key signaling pathway in response to drought, ABA content in crops increases with exposure to water deficits, which causes the closure of stomata on leaves to reduce water loss. ABA-activated kinases SnRK2s mediate the phosphorylation of the plasma membrane NADPH oxidase RhohF, which generates O^2−^ in the apoplastic space simultaneously. The generated O^2−^ can then form H_2_O_2_ as a signaling molecule to regulate stomata closure. Activated SnRK2s could phosphorylate the ion channel SLAC1, which also mediates ABA-induced stomatal closure and reduces transpirational water loss under drought [147]. Peptide CLE25 can sense water shortage in the root area and transmit the stress signal long-distance to the leaves through the vascular bundle by binding protein receptors BAM1 and BAM3 to mediate the expression of *NCED3*, causing an increase in ABA content while reducing the stomatal conductance and effectively improving root drought resistance [148]. When sensing the continuous drying of soil, more roots would generate chemical signals, including ABA, to regulate the growth and development of the upper part of the plant based on the water availability [149]. Although various plant hormones regulate this process, ABA is the one that has more-convincing studies. Then, the physiological traits (e.g., stomata conductance) of leaves are regulated to change the ratio of roots to leaves. It has been reported that the concentration of ABA in xylem and the content of ABA in roots have an approximately linear relationship, showing that xylem ABA concentration can be a quantitative indicator of root-generated ABA, which reflects the sensing ability of roots to soil conditions [23,24]. This type of root-generated signal could largely reduce water loss though stomata adjustment before leaf tissue senses the water deficit. It could be the first barrier of drought resistance in plants. As the stress continues, elder and lower leaves start to wilt, which could be the second barrier of drought resistance in plants [25]. The root–shoot signaling sets a theoretical basis for improving WUE through sensing water availability in the root area. Further study would provide more theoretical support for further improvement in water management and WUE by ABA in the field.

### 4.2. ABA Functions in Crop Roots

Other than the root–shoot communication function, ABA participates in the regulation of growth and development of the root cells. Roots display hydrotropism in response to moisture gradients, which is the pivotal mechanism directing root growth for water acquisition. ABA is important for root hydrotropic responses by regulating the action of transition and elongation zones where differential growth responses occur in the root apex [26]. This type of hydrotropism relies on the PYR/PYL/RCAR-PP2Cs-SnRK2s pathway, which is the core of ABA signaling transactions [26,150,151]. As a key gene regulating root growth as discussed above, the *MIZ1* gene interacts with ABA signaling in the cortex to mediate root hydrotropism response [26,152]. In maize, ABA accumulation can contribute to the maintenance of maize primary root growth under drought [153]. Particularly, ABA is associated with growth maintenance in the apical region of the growth zone [154]. In soybean, ABA accumulation also occurs in roots under water-deficit conditions and is enhanced in a *PgTIP1* transgenic line [27]. In wheat root, ABA regulates the premature differentiation of root apical meristem under drought [155]. Several rice genotypes also exhibit enhancement or stability in root length in response to ABA treatment [156]. ABA also regulates ROS generation and thus directs the protection of plant tissues from oxidative damage under stress conditions [157]. It can also interact with H_2_O_2_ to induce the activity of cytosolic glucose-6-phosphate dehydrogenase, an important enzyme in the ascorbate–glutathione cycle under drought in soybean roots [158]. Moreover, *ABP9*, encoding a bZIP transcription factor from maize, may participate in the ABA signaling pathway by altering the expressions of multiple stress-responsive genes in transgenic cotton lines, which improved the root biomass and the survival rates of two *ABP9* overexpression lines compared with the wild-type under drought conditions [159].

### 4.3. Interactions between ABA and Other Hormones in Crop Roots

Multiple other plant hormones can interact with ABA to regulate the growth and responses to drought stress of roots. It has been reported in *Arabidopsis* that ABA regulates root growth under osmotic stress conditions via an interacting hormonal network with auxin and ethylene [29]. However, interactions of ABA and other hormones in crop roots under drought conditions have not been developed as a complete network. Most studies are limited to the interaction of ABA with one other hormone. ABA interacts with auxin in the root tip of rice to modulate auxin transport, which enhances proton secretion for maintaining root growth under water-deficit conditions [160]. In barley, ABA accumulation can promote auxin response in roots, resulting in enhanced rhizosheath formation under drought conditions [161]. Ethylene interacts with ABA in the primary root region of growth maintenance in maize under water-deficit conditions. The accumulation of ABA limits the production of excess ethylene to ensure the growth of primary roots [162]. During seed germination, gibberellins (GA) and ABA are antagonistic in regulating the emergence of the primary root [163]. In rice root systems, Lin et al. [164] reported that high levels of GA can activate APC/CTE, a ubiquitin ligase complex, to promote the degradation of the rice root growth regulation gene *OsSHR1*, thereby controlling the growth of the root. During this process, application of moderate-level ABA could antagonize GA to maintain root growth. In cotton roots, GA levels have been observed to decrease while ABA levels rise under drought conditions [30]. Jasmonate (JA) could stabilize ABA levels in roots under water-deficit conditions [165], and ABA level reduction is observed in JA-deficient plant roots [28]. Recent studies on *Arabidopsis* suggested that PM H^+^ extrusion is crucial for primary root growth and the hydrotropic response in roots, which is regulated by ABA [71] and brassinosteroid [166,167]; this could be an interesting interaction to investigate in crop roots.

As summarized above, ABA regulates root growth in various crops, and its functions in signaling are essential for root–shoot communication under drought conditions. It is also clear that, similar to the genes regulating root growth mentioned above, most molecular mechanistic studies have not been well-developed in crops. Future studies should not only aim to understand how internal or external ABA changes root traits as correlated phenomena, but also focus on the molecular mechanisms of how ABA regulates specific compounds in roots and how ABA interacts with other plant hormones under drought conditions (Figure 5).

## 5. Nutrient Regulations on Crop Root Responses to Drought Stress

The physiological and metabolic responses of crop root system growth and architecture to drought stress are regulated by nutrients. Although a recent review [168] demonstrated the influence of water status on nitrogen regulation in *Arabidopsis* and rice at the whole-plant level (for root traits, only root branching was mentioned), the understanding of how water deficiency affects other nutrients (e.g., phosphorus, potassium, and sulfur) and how this interaction contributes to root growth needs further summary. Here, we review studies on the interaction of water deficiency with various nutrients and the effects on crop root growth (Table 1).

### 5.1. Nitrogen

Studies have shown that nitrogen can enhance resistance to drought stress by changing root structures [31]. Under water-deficit conditions, appropriate nitrogen application can affect the root development of cotton by increasing the root–shoot ratio, root density, lipid peroxidation, and antioxidative enzyme activity [169,170,171]. High nitrogen application can significantly promote construction of the root system and enlarge root thickness and root system volume under drought conditions in both sensitive and tolerant wheat cultivars [172]. One possible reason for the synergistic effect of nitrogen and water is that nitrogen can change the hydrodynamic properties of root cell membranes by changing the concentration of cellular nitrates (NO_3_^−^), resulting in water potential gradients. Such gradients increase water movement from soil into root cells [173,174]. This may also be related to aquaporins, since aquaporins are differentially expressed in plant roots in response to nitrogen availability. In rice, nitrogen supply can increase the expression of root-specific aquaporin genes *OsPIPs* and *OsTIPs*. However, the expression of these genes does not decrease during nitrogen deprivation. These expressions are correlated with root hydraulic conductivity [175]. Such a mechanism may be regulated by the NRT2.1 nitrate transporter, as it was found to impact the transcript abundance of PIP aquaporins in *Arabidopsis* that also correlated with root hydraulic conductivity [176]. It has also been reported that, compared with nitrate, ammonium (NH_4_^+^) can better improve the drought resistance of rice seedlings. Due to the accumulation of ROS, drought-induced oxidation damage in roots with NO_3_^−^ treatment was significantly higher than that with NH_4_^+^ treatment. Simultaneously, the activity of antioxidative enzymes was increased in roots with NH_4_^+^ treatment [177]. In addition, drought-mediated deep-root effects (e.g., *DRO* gene-mediated) increased water absorption while also increasing nitrogen absorption efficiency in a variety of crops, including wheat and sorghum, resulting in the effect of water–nitrogen coupled absorption [178,179,180,181].

### 5.2. Phosphorus

As another major macronutrient, phosphorus also plays significant roles in regulating the growth of crop roots under drought. For soybean varieties, moderate phosphorus application can improve yield, accumulation of phosphorus and nitrogen in roots, and daily water consumption, while a high phosphorus fertilizer rate may decrease these traits, indicating that there is an interaction between WUE and phosphorus application. Soybean varieties with larger numbers of adventitious roots and lateral roots per unit length have better phosphorus absorption ability [182]. In some other legume plants, genotypes that have better adaptation to low-phosphorus environments tended to have a shallower basal root angle in order to acquire phosphorus that was mostly concentrated in the top layer of soil, whereas other genotypes that have better adaptation to late growth-stage drought stress tended to form deeper roots for uptake of water resources existing in deeper layers of soil. For the phosphorus-acquiring genotypes, the root system reduced the respiratory burden of root growth and changed root-hair formation to increase phosphorus acquisition. Root architectural patterns in these genotypes enhanced topsoil foraging and phosphorus acquisition but appeared to incur tradeoffs in water acquisition and spatial competition [183]. As for wheat, deep placement of phosphorus fertilizer enhanced growth by promoting deep root development [184]. Wheat also displayed different phosphorus acquisition efficiencies across genotypes, which is associated with enhanced expression of the phosphorus transporter *TaPht1* genes. As a result, genotypes with higher phosphorus acquisition efficiency presented higher biomass production and enhanced root development, root–shoot ratio, and root efficiency for phosphorus uptake under phosphorus-deficient conditions [185]. In cotton, the application of phosphorus fertilizer under water-deficit conditions promoted phosphorus efficiency ratio, phosphorus absorption efficiency, and phosphorus transfer efficiency in roots. Under such a condition, the biomass and the yield of cotton were increased, showing an enhancement to drought resistance by phosphorus fertilization [186]. These studies show that the amount of phosphorus resources in soil and the absorption efficiency, especially under drought conditions, affect the growth traits and RSA. This process also involves the balance between water and nutrients.

### 5.3. Potassium

As a key cation element in most plants, potassium increases cell membrane stability, positively regulates osmotic adjustment, maintains the activity of aquaporins, and enhances water uptake, which may contribute to the promotion of root growth [187]. Studies indicated that potassium deficiency may promote the activity of NADPH oxidase to accumulate ROS content in legume root cells. This process is related to ABA accumulation, and it may be relieved by potassium fertilization [187,188]. In maize, proper application of potassium fertilizer may promote root growth by increasing the root surface area and water uptake [189]. In rice, molecular evidence has shown that several genes are involved in improving drought tolerance by promoting the acquisition and transport of potassium in roots. The overexpression of *OsAKT1*, a potassium inward rectifying channel, increased potassium content in the root tissue, and thereby improved the drought tolerance of rice [190]. A high-affinity potassium transporter gene, *OsHAK1*, was reported to be specifically expressed in roots under drought conditions and to promote potassium transport and acquisition as well as grain yield of rice. It could also positively regulate the expression of other drought-response genes, including *OsAKT1* and *OsTPKb*, two genes that regulate the homeostasis of potassium in cells [191]. Additionally, another rice gene, *RAA1* (Root Architecture Associated 1), participated in the auxin-mediated root development and could be enhanced by *HAK1*. Transgenic *RAA1* plants with inserted *HAK1* enhancers displayed promoted root system size and increased potassium, proline, and ABA contents under drought conditions compared with wild-type [192]. These transgenic studies reported positive effects of potassium transport genes on yields. Therefore, potassium transport genes could be considered a key factor for promoting plant drought resistance through root traits in molecular breeding.

### 5.4. Sulfur

Sulfur is another essential macronutrient for plant growth and development. Current studies of sulfur in crop roots are relatively less-focused than the macronutrients described above. Multiple compounds in the sulfur metabolic pathways, especially sulfur-containing amino acids and their derivatives, are critical for plant health and growth [193,194]. Sulfur metabolism not only plays a key role in the primary metabolism of plants, but also provides the structural components of essential cellular molecules. Some of the metabolites in sulfur metabolism pathways are important for plant acclimation to stressful environments [195]. The distribution of sulfate apparently shifts to maize roots, resulting in a significant increase in thiols produced by the assimilation of sulfates in the roots, and drought may affect the transcription of the membrane-anchored sulfate transport system in the root system [34]. A major function of sulfur in response to water-deficit stress in roots lies in the production of two sulfur-containing amino acids, cysteine and methionine, and their derivatives [196]. As mentioned above, glutathione, an important antioxidant, is derived from cysteine. The importance of cysteine to drought stress resistance is mostly achieved through glutathione functions. Along with its antioxidative function, glutathione is also involved in the detoxification of heavy metals [197,198], transfer and storage of sulfur [199], regulation of expression of defense-related genes [200], and protein activity [201] under stressed conditions. However, the regulation of glutathione metabolism may be different in primary roots of different crops (cotton and maize) in response to drought [37]. Similar to the relationship between cysteine and glutathione, the importance of methionine in responses to water-deficit stress is mostly achieved through a derivative, S-adenosylmethionine (SAM). SAM is a multi-functional amino acid derivative. It plays important roles in regulating plant development, stress responses, and metabolite accumulation [202,203]. SAM is one of the precursors of ethylene, which is an important plant hormone that can interact with ABA to regulate root growth, as mentioned above, as well as a precursor for polyamines, which play an important role as osmoprotectants during drought [204]. Polyamine metabolism is processed through the methionine salvage pathway (Yang cycle), which is regulated by the availability of sulfur in the plant cell [205,206]. Additionally, polyamines can interact with ABA [207] and ROS [208,209] under water-deficit conditions. Several studies on crop roots found that SAM synthetase (SAMS) was induced by drought stress in soybean [210,211] and wheat genotypes with better drought tolerance [212].

**Table 1:** Interactions of roots and nutrients in crops under drought stress.

Species	Nutrient	Interaction	References
**Cotton**	nitrogen	Nitrogen can enhance the root–shoot ratio, root density, lipid peroxidation, and activity of antioxidative enzymes	[169,170,171]
**Cotton**	phosphorus	Phosphorus fertilizer enhances stress resistance in cotton and significantly increases biomass and yield	[186]
**Legume**	phosphorus	Deeper roots for uptake of water resources existing in deeper layer of soil rather than acquiring phosphorus	[183]
**Maize**	potassium	Potassium fertilization may promote root growth by increasing the root surface and water uptake	[189]
**Maize**	sulfur	SAM synthetase is induced by drought stress	[210,211]
**Rice**	nitrogen	*DRO1* regulates nitrogen and water uptake, forming deeper roots and improving grain productivity	[181]
**Rice**	nitrogen	Aquaporin gene expression correlates with nitrogen supply and deprivation	[175]
**Rice**	potassium	Root-specific overexpression of a potassium inward rectifying channel, OsAKT1, improves drought tolerance	[190]
**Rice**	potassium	Root-specific expression of the potassium transporter OsHAK1 can promote potassium acquisition and grain yield	[191]
**Sorghum**	nitrogen	Root diameter and root length density can improve water uptake and positively regulate nitrogen uptake	[178]
**Soybean**	phosphorus	Root traits can contribute to high nutrient uptake efficiency and benefit yield	[182]
**Soybean**	sulfur	SAM synthetase is induced by drought stress	[212]
**Wheat**	phosphorus	Deep placement of phosphorus enhanced growth by promoting deep root development	[184]
**Multiple crops**	nitrogen	Growing roots seeking deeper water resources can assist with nitrate uptake	[179]

Roots play an integral role in the acquisition of water and nutrients in crops. This role needs to find a balance between water and nutrients. According to the distribution of nutrients and water in different soil layers, the crop root system can better obtain nutrients and water by adjusting its formation and density to find a balance between water and nutrient absorption and to improve the absorption efficiency. The studies discussed above mainly explored the regulatory role of single nutrients in root growth under drought. More attention should be focused on the coupling of multiple nutrients and their regulatory effects on root growth under drought. Precisely quantifying the contribution of different nutrients to the drought resistance of crop roots needs to be studied. As a goal, we aim to provide a more useful and efficient scientific basis for the integrated regulation of water and nutrients in crop roots under drought conditions in the future.

## 6. Crop–Microbial Interactions and Regulations in the Rhizosphere under Water-Deficit Conditions

The rhizosphere is the area where strong interactions occur among crop roots, soil, and microorganisms. Root system and rhizosphere microbes affect each other through interactions (Figure 1). Changes in root growth and architecture affect the composition and distribution of microbiomes, while, in turn, microbes in the rhizosphere significantly impact the development and growth of root systems [213]. Recent findings suggest that duration of drought substantially influences the composition and activity of plant root-associated microbiomes [214,215,216]. Rhizosphere symbiotic microorganisms are beneficial to the host crops by improving root architecture and absorption of water and nutrients. Here, we are more focused on how rhizosphere microbes, including bacteria, rhizobia, plant growth-promoting rhizobacteria (PGPRs), and arbuscular mycorrhizal fungi (AMF), improve the drought resistance of roots and regulate RSA in crops.

Studies have shown that soil bacterial co-occurrence networks are less stable than fungal networks in response to drought [217]. Further work displayed that drought-induced shifts in bacterial composition was characterized by an almost universal increase in monoderm bacteria and a decrease in diderm lineages [218]. These characteristics indicate that rhizosphere microbes have different tolerances to drought stress, which, in turn, affects the connection between crop roots and the rhizosphere. Carbohydrates and amino acids, the well-known components of root exudates that have been shown to support the growth of rhizobacteria, are the likely candidates that could promote monoderm growth, since these plant primary metabolites increased in response to water-deficit stress in crop roots as mentioned above. Among various classes of compounds, it is interesting to note that the glycolysis intermediate, glycerol-3-phosphate, which is used to produce teichoic acid, a precursor necessary for peptidoglycan biosynthesis and cell-wall formation of bacteria [219], accumulated at a significantly high level during water-deficit stress in sorghum roots, plausibly enabling preferential root colonization by monoderm bacteria, which then aided in drought tolerance [216].

Manipulation of the rhizosphere microbiota is an effective strategy to confer drought tolerance and improve nitrogen use in management practice [218,220,221]. For example, the monoderm genus *Streptomyces* offers potential contributions to improve plant fitness under water-deficit stress. Along drought gradients, Fitzpatrick et al. [222] observed that plant species with greater drought-induced increases in endosphere *Streptomyces* had greater drought tolerance. In crops, the colonization of sorghum seedlings with *Streptomyces* isolates increased the root growth under drought, but no obvious stimulating effect was found in well-watered conditions [216]. Likewise, the root-associated bacterium *Variovorax paradoxus* 5C-2, which contains the ACC deaminase that degrades the ethylene precursor 1-aminocyclopropane-1-carboxylic acid (ACC), which is also an important module in sulfur metabolism [196], was reported to increase the growth, yield, and WUE of pea plants grown in drying soil [223].

Other than bacteria, the abundance of AMF may also help the host plant increase drought tolerance by enhancing antioxidant enzyme activity, which reduces oxidative stress with various strategies as highlighted above, and thus improves WUE and biomass accumulation [224]. AMF is also considered to increase the water arability of crops through external hyphae and glomalin to increase crop resistance to drought stress [225]. Studies also showed AMF could regulate RSA to promote the growth and development of a root system in citrus and maize in arid soil [226,227]. However, the effects of AMF interaction with other rhizosphere microorganisms on the RSA of crops (synergy or competition) under different water-deficit conditions, and by which mechanisms these effects work, require further study [213].

PGPRs affect RSA mainly through secreting abundant metabolites to regulate developmental signals in crop root systems. These effects regulate RSA by changing the growth traits of primary roots, lateral roots, and root hair, which are the most important components of the root system, as illustrated above. Studies have demonstrated that PGPRs can promote or inhibit the elongation of the primary root. However, lateral roots and root hair were significantly promoted by PGPRs, with increased biomass of the whole root system of chickpeas [228]. In order to utilize PGPRs and their metabolites to regulate the formation of ideal RSA in different crops and to promote the absorption and utilization of the rhizosphere water resource, it is still necessary to systematically explore new signaling substances for PGPRs to regulate RSA to reveal their underlying mechanisms, and to promote the application of PGPRs in crop drought resistance [213].

In fact, as plant fitness is interconnected with the composition and activity of the phytobiome [229], harnessing rhizosphere microbiomes under drought may have profound consequences for crop yields, soil carbon sequestration, and nutrient cycling. Studies have been designed to characterize microbially mediated strategies for enhancing water and nutrient acquisition [218,221], a promising approach to improve food security in undeveloped and developing countries and to develop sustainable agriculture in developed nations by reducing intensive fertilization and irrigation. However, most of these findings are lab-based without sufficient evidence related to rhizospheres in the field. Future studies on plant–soil microbial interactions in the rhizosphere under water-deficit conditions should focus on finding how microbes and roots interact in response to drought in real natural conditions and how to apply rhizosphere microbiomes in the field [230]. Studies should be further expanded to the complex of crop, root, rhizosphere, microbiome, and soil [231]. We expect to demonstrate the regulatory mechanism of rhizosphere microbes to the responses of crop roots to water-deficit stress by integrated technologies, including in situ field isotope abundance measurement, zymography, computed tomography (CT) scan of the root–soil layer, isotope-ratio mass spectrometry, root secretion metabolomics, and microbiomics. Thus, we can develop better strategies for improving the resistance of crop roots to drought stress as well as water and nutrient use efficiency through regulation by rhizosphere microorganisms.

## 7. Conclusions and Perspectives

In summary, this article reviewed both classic and recent research efforts regarding root responses to drought in crops, particularly emphasizing molecular mechanisms to regulate root growth in response to water-deficit stress and interactions with nutrients and soil microbiota. In an era of continuous climate change worldwide, the importance of root response to drought stress has been acknowledged and has been studied with different focuses and technologies. Traditional approaches to root trait screening and metabolism analysis are still important to evaluate drought tolerance and develop breeding strategies for certain crops. With the advances in cytology, biochemistry, and molecular biology, research on the molecular mechanisms of crop root responses to water-deficit stress has made rapid progress. A large number of related genes have been identified and manipulated, while many important regulatory pathways have been discovered [232]. “Omics” studies (including genomics, transcriptomics, proteomics, metabolomics, and phenomics) provide more-efficient methods for identifying key factors in roots that are essential for plant growth and fitness under gradually severe drought conditions [35,37,125]. Advanced molecular manipulation technologies are also utilized to improve crop adaptation in water-stressed environments [26,81]. It is anticipated that researchers will be able to apply molecular findings to breeding methodologies and technologies aimed at improving the drought resistance of crop roots. In recent years, based on the development of biomarkers using a few important genes to assist breeding, integrated multi-omics has also provided a platform for developing a crop whole-genome selection model using an AI-optimized algorithm. Additionally, haploid breeding and targeted gene editing technologies provide the basis for selecting multiple traits and imposing multiple genetic loci with low effects at the same time. Thus, the accuracy and efficiency of crop breeding can be improved [232].

The responses of major crop roots to drought stress involve complex biological processes. Moreover, current research mainly focuses on the effects of a single stress factor to crop roots, while roots can be suffering multiple stress factors, including water, nutrients, and microbes, in actual field conditions. Therefore, how to sense and respond to different stress factors and whether there are any commonalities among the responses to various stress factors that can be integrated for analysis remain key aspects in current studies. Future studies need to focus on connecting gene expression, protein regulation, metabolite accumulation, and nutrient fertilization with growth traits and phenotypes of the crop roots under water-deficit conditions to build up a network including responses in multiple aspects (e.g., plant hormone crosstalk, water and nutrient interactions, and plant–soil microbial feedback) for a more comprehensive understanding of the mechanisms of drought tolerance in crop roots. This network can give a guide on the important traits for improving root adaptation to drought stress, which can be enhanced by breeding strategies. In the near future, it is expected that the drought resistance of major crop roots (e.g., maize, rice, wheat, soybean and cotton) will improve with integrated technologies. Development of a series of new germplasms and genotypes with better tolerance to water-deficit conditions and future climate change as products of advanced breeding strategies is also anticipated. Together, studies focusing on uncovering the mechanisms of crop roots in response to drought stress in the rhizosphere will improve our knowledge of plant stress physiology and provide insight for breeding strategies and agronomy to produce high-yield and drought-tolerant crops.

## Data Availability

Not applicable.
